# Bioinspired piezoelectric patch design for sonodynamic therapy: a preclinical mechanistic evaluation of rotator cuff repair and functional regeneration

**DOI:** 10.3389/fbioe.2025.1565347

**Published:** 2025-05-21

**Authors:** Rui Shi, Fei Liu, Qihuang Qin, Pinxue Li, Ziqi Huo, You Zhou, Chunyan Jiang

**Affiliations:** ^1^ Beijing Jishuitan Hospital, Capital Medical University, Beijing, China; ^2^ Beijing Research Institute of Traumatology and Orthopaedics, Beijing, China

**Keywords:** rotator cuff, tendon-bone interface, electrospinning, biomimetic piezoelectric patch, sonodynamic therapy

## Abstract

**Indroduction:**

The rotator cuff tendon-bone interface exhibits a gradient histological composition, including graded mineral content and interwoven collagen fibers. Following rotator cuff injury repair, the lack of a compositional, structural, and functional gradient at the interface results in stress concentration and a high rate of postoperative re-tears. Piezoelectric materials, known for modulating cellular functions and promoting stem cell proliferation and differentiation, have garnered increasing attention in tissue repair applications.

**Methods:**

In this study, a biomimetic piezoelectric patch with progressive compositional and structural variations was designed and fabricated. The patch, composed of gelatin/PLGA/nHA/BTO, integrates aligned and random fiber structures. The aligned layer mimics the tendon-side structure of the rotator cuff tendon-bone interface, while the random layer replicates the bone-side structure.

**Results:**

The bioinspired patch exhibits excellent biocompatibility. The piezoelectric signals generated under ultrasound stimulation can induce osteogenic and tenogenic differentiation of stem cells, as well as regulate M2 polarization of macrophages, thereby promoting the repair and regeneration of supraspinatus tendon injury in a rabbit model of rotator cuff injury.

**Discussion:**

This study highlights the potential of biomimetic piezoelectric patches in orthopedic rotator cuff repair and offers new possibilities for developing advanced materials to regenerate the rotator cuff tendon-bone interface.

## 1 Introduction

Rotator cuff injuries are among the most common musculoskeletal disorders. Data indicate that the prevalence of rotator cuff injuries ranges from 5% to 39%, with a significantly higher incidence observed in individuals over the age of 60 and in those engaged in repetitive high-intensity shoulder joint activities over prolonged periods (McElvany et al., 2015; Rothrauff et al., 2017). Rotator cuff tears or injuries often result in persistent shoulder pain and restricted mobility. In severe cases, surgical intervention is required to reattach the tendon to the humerus. However, rotator cuff tears frequently exhibit poor healing, and the recurrence rate following surgical intervention remains high. The retear rate after rotator cuff repair is estimated to be as high as 94%. Consequently, rotator cuff injuries continue to pose a longstanding challenge in orthopedic medicine (Lee et al., 2016).

The tendon-to-bone interface of a normal rotator cuff exhibits a unique transitional zone comprising tendon, unmineralized fibrocartilage, mineralized fibrocartilage, and bone. Each region is characterized by specific cell types and distinct extracellular matrix compositions, demonstrating progressive variations in tissue composition, structure, and mechanical properties (McElvany et al., 2015; Angeline and Rodeo, 2012; Wu et al., 2017; Wang et al., 2006; Rossetti et al., 2017; Apostolakos et al., 2014; Benjamin et al., 2002; Shaw and Benjamin, 2007; Smith et al., 2012). This complex gradient tissue structure results in a gradual transition in mechanical properties, enabling load transmission and reducing stress concentration, thereby maintaining the function and homeostasis of the tendon-to-bone interface. In the aftermath of rotator cuff injury, a combination of diminished cell density and inadequate vascular supply impedes the synthesis of the extracellular matrix, thereby inducing a protracted healing process. The repaired interface often develops scar tissue rich in type I collagen (Patel et al., 2018), which compromises biomechanical properties and increases the risk of retear (Angeline and Rodeo, 2012).

Currently, various biomaterials have been utilized to design biomimetic scaffolds with gradient structures and functions resembling the rotator cuff. These scaffolds aim to enhance the repair strength of the tendon-to-bone interface and promote *in situ* regeneration of rotator cuff tissue (Lin et al., 2018). Decellularized tendon/fibrocartilage scaffolds exhibit excellent biodegradability, high biocompatibility, low immunogenicity, and a close resemblance to the structure and composition of the extracellular matrix, making them suitable for enhancing tendon-bone healing at the rotator cuff interface (Chen et al., 2020; Lu et al., 2019; Ning et al., 2015). Kim et al. (2014) constructed a four-layer tendon-to-bone structure scaffold using different biomaterials, including collagen, collagen with chondroitin sulfate, collagen with low-calcium HA, and low-calcium HA. The study highlighted that scaffold composition and structure, particularly the mineral content gradient, are critical parameters influencing scaffold performance and biological behavior. Sun et al., 2016 incorporated collagen and nano-hydroxyapatite into PLGA fibers and PCL fibers, respectively, to fabricate co-electrospun PLGA-PCL dual nanofiber scaffolds, which demonstrated enhanced collagen integration and osteogenic effects. Chen et al. (2019) processed natural fibrous cartilage tissue into a book-shaped structure, creating a book-shaped acellular scaffold with excellent cell loading capacity and *in vitro* chondrogenic induction potential. Based on the heterogeneous structure and composition of the rotator cuff tendon-to-bone interface, Liu et al. (2019) and Tang et al. (2020) developed a gradient book-shaped decellularized tri-phase (bone-fibrocartilage-tendon) scaffold, which demonstrated enhanced osteogenic, chondrogenic, and tenogenic inductive capacities. However, its clinical application is limited by stringent processing requirements and the need to establish secondary injuries during its preparation. Dai et al. (2024) developed a biomimetic gradient-structured scaffold incorporating type I collagen (COL1) and hydroxyapatite (HAp) loaded with human amniotic mesenchymal stem cells (hAMSCs), which demonstrated enhanced capacity to orchestrate stem cell lineage commitment and facilitate interfacial tissue regeneration. Zhang Y. et al. (2024) engineered a three-dimensional biomimetic chitin scaffold leveraging the multiscale structural hierarchy and surface bioactivity of organic matrices derived from cuttlefish bone, demonstrating enhanced mechanobiological competence for tendon-bone interface reconstruction. However, the absence of preclinical validation in rotator cuff injury models necessitates systematic evaluation of its spatiotemporal therapeutic efficacy, particularly regarding long-term fibrocartilage regeneration and biomechanical integration under physiological loading conditions. Current research on biomimetic scaffolds mainly focuses on mimicking the structure of the rotator cuff, but it has not given sufficient attention to the various physiological signals required for tendon-bone interface repair and regeneration, including biochemical, electrical, and mechanical signals.

Piezoelectric materials are a class of materials that generate an electric charge (or voltage) when subjected to external forces or undergo deformation when an electric field is applied. The piezoelectric effect in these materials arises from the asymmetric arrangement of internal electric dipoles, leading to a redistribution of charge under external stress, resulting in the formation of surface charge. The mechanical forces required for the self-activation of piezoelectric biomaterials can be derived from the interactions between cells and scaffolds or from the physiological activities of the organism (Yang et al., 2021; Chen et al., 2021; Xia et al., 2022; Liu et al., 2021a). Additionally, external stimuli such as ultrasound (Font et al., 2015; Dolai et al., 2022) can controllably activate piezoelectric materials in a wireless and non-invasive manner. Therefore, piezoelectric materials have the potential to mimic the mechanoelectric energy conversion systems found in rotator cuff tissues. Currently, most related studies are focused on bone, cartilage, neural, and other tissues (Liu et al., 2023; Nain et al., 2024; Shlapakova et al., 2024; Wu et al., 2024). The tendon-bone interface of the rotator cuff contains a transitional zone from bone to tendon, exhibiting distinct piezoelectric properties (Liu et al., 2021b). Piezoelectric materials can reconstruct the piezoelectric network at the tendon-bone interface, promoting tendon-bone healing. Liu et al. (2023) incorporated antioxidant polydopamine-modified HA and BaTiO3 into a biodegradable PHBV polymer matrix. Under piezoelectric stimulation and the action of bioactive components, the biomimetic piezoelectric periosteum exhibited excellent biocompatibility, osteogenic activity, and immunomodulatory functions *in vitro*. Baumgartner et al., 2022 fabricated a piezoelectric core-shell structure (i.e., a structure composed of a polydimethylsiloxane (PDMS) core and a piezoelectric polyvinylidene fluoride (PVDF-HFP) shell) using coaxial electrospinning. Their study demonstrated that tendon differentiation is influenced not only by the morphology of nanofibers but also by dynamic mechanical stimuli and the associated electrical signals generated. Zhang Q. et al. (2024) designed and constructed a dual-sided nanofiber scaffold, with one side composed of poly (L-lactic acid)/zinc oxide (PLLA/ZnO) fibers and the other of poly (L-lactic acid)/barium titanate (PLLA/BTO) fibers. This scaffold exhibited outstanding piezoelectric properties, effectively converting mechanical forces into electrical signals during movement. It demonstrated excellent potential for tendon and bone repair while promoting cell growth and differentiation through electrical stimulation. Huang et al. (2025) developed a novel Janus asymmetric piezoelectric adhesive hydrogel that effectively promotes tendon-bone interface healing under ultrasound irradiation by leveraging anisotropic charge distribution and mechanoelectrical coupling. Building on the “muscle–electrical signal coupling” paradigm, Li X. et al. (2025) engineered an injectable piezoelectric hydrogel PVA/CNF/BTO@PDA for interfacial regeneration. However, achieving spatiotemporally controlled *in vivo* hydrogel deformation to generate localized electrical microcurrents with therapeutic precision remains a critical challenge. To date, no prior studies have reported the integration of piezoelectric materials into biomimetic scaffolds for rotator cuff repair. Motivated by this technological gap, we pioneered the synergistic combination of piezoelectric composites with anisotropic scaffold architectures, establishing a novel therapeutic modality that leverages ultrasound-activated electromechanical coupling for sonodynamic augmentation of tendon-bone interface regeneration.

In this study, we developed a biomimetic piezoelectric patch with a compositional and structural gradient, Gelatin/PLGA/nHA/BTO, using electrospinning technology. This patch incorporates both aligned and non-aligned fiber structures to simulate the compositional and structural gradients of the tendon-bone interface. Combined with *in vitro* ultrasound stimulation, the patch reconstructs the piezoelectric network at the injury site to promote tendon-bone integration. Biodegradable PLGA, known for its biocompatibility, is a commonly used material in tendon engineering and has demonstrated effectiveness in repairing rotator cuff injuries (Ruiz-Alonso et al., 2021). The natural polymer gelatin enhances hydrophilicity and cell compatibility (Guillén-Carvajal et al., 2023). Nano-hydroxyapatite (nHA), which is analogous to the inorganic components of bone tissue, has been shown to exhibit excellent biocompatibility and osteoconductivity. It is widely recognised as a leading bone substitute material (Angeline and Rodeo, 2012; Hoveidaei et al., 2024; Lowe et al., 2020). Barium titanate (BTO), a piezoelectric ceramic, has been demonstrated to show good biocompatibility, bioactivity, and osteogenic potential. The piezoelectric potential of BTO promotes apatite deposition, cell differentiation, and bone formation (Wu et al., 2023; Khare et al., 2020; Swain et al., 2023). Both *in vitro* and *in vivo* studies have shown that the biomimetic piezoelectric patch, Gelatin/PLGA/nHA/BTO, exhibits excellent piezoelectric properties and biocompatibility. Under ultrasound stimulation, it facilitates the repair of the rotator cuff tendon-bone interface and modulates the inflammatory environment to further promote tissue regeneration. This study provides strong experimental evidence for the development of novel electrically stimulated scaffold materials for tendon-bone repair, offering significant theoretical insights and potential applications.

## 2 Methods

### 2.1 Preparation of biomimetic rotator cuff patches

Pure PLGA Solution: A total of 2.780 g of PLGA was weighed into a dry sample vial, followed by the addition of 10 mL hexafluoroisopropanol (HFIP) solution. The mixture was stirred using an LC-UMS-6 magnetic stirrer until a homogeneous solution was obtained, resulting in an electrospinning solution with a PLGA mass concentration of 15%.

PLGA/Gelatin Solution: A total of 0.309 g of gelatin was weighed into a dry sample vial, followed by the addition of 10 mL HFIP solution. The mixture was subjected to ultrasonic treatment for 30–60 min until the gelatin was fully dissolved, after which 2.780 g of PLGA was added. The vial was placed on a thermostatic magnetic stirrer and stirred until a homogeneous solution was formed, resulting in an electrospinning solution with a PLGA-to-gelatin mass ratio of 9:1.

PLGA/nHA Solution: A total of 0.185 g of nano-hydroxyapatite (nHA) was weighed into a dry sample vial, followed by the addition of 10 mL HFIP solution. The mixture was subjected to ultrasonic treatment for 30–60 min to ensure uniform dispersion of nHA, after which 2.780 g of PLGA was added. The vial was placed on a thermostatic magnetic stirrer and stirred until a homogeneous solution was formed, resulting in an electrospinning solution with a PLGA-to-nHA mass ratio of 15:1.

BTO Solution: A total of 1.300 g of nano-barium titanate (BTO) was weighed into a dry sample vial, followed by the addition of 10 mL of anhydrous ethanol. The mixture was subjected to ultrasonic treatment for more than 60 min to achieve uniform dispersion, resulting in an electrospinning solution with a BTO mass concentration of 7%.

Electrospinning Process: The above-prepared solutions were combined and processed using a dual-nozzle electrospinning setup. After achieving a stable electrospinning process, the fibers were continuously collected for 8 h. The resulting electrospun fiber membranes were air-dried for 24–48 h to remove residual HFIP solvent from the membrane surface.

### 2.2 Characterization of biomimetic rotator cuff patches

The microstructure of the biomimetic rotator cuff patches was observed using a scanning electron microscope (SEM, S-4800). The chemical structure of the patches was analyzed using a Fourier transform infrared spectrometer (FTIR, Bruker Tensor-37). The phase structure of the patches was characterized with an X-ray diffractometer (XRD-6000, Shimadzu). Energy-dispersive spectroscopy (EDS) was performed using the SEM (S-4800) equipped with an EDS attachment. The swelling ratio of the patches was evaluated by measuring their water absorption capacity. The static contact angle of different rotator cuff patches was determined using a static contact angle analyzer (SL200A). The mechanical properties of the patches were characterized using a universal testing machine (TSC1000M).

The piezoelectric constants (d33) of various rotator cuff patches were measured using a quasi-static d33 tester (ZJ-3A, Institute of Acoustics, Chinese Academy of Sciences). The open-circuit voltage and short-circuit current of the patches were measured using an electrometer (Keithley 6517B). The piezoelectric properties of the biomimetic piezoelectric rotator cuff patch (Gelatin/PLGA/nHA/BTO) were characterized using a Bruker Multimode 8 atomic force microscope.

### 2.3 Cellular experiments

#### 2.3.1 Cultivation of bone marrow mesenchymal stem cells (BMSCs)

The BMSCs were obtained from iCell Bioscience Inc. (Shanghai, China). The third-generation rat BMSCs were seeded into T25 culture flasks or plates containing DMEM supplemented with 10% fetal bovine serum (FBS) and 1% penicillin-streptomycin. The cells were cultured at 37°C with 5% CO2.

#### 2.3.2 *In Vitro* cell proliferation assay

Cell proliferation on the scaffolds was evaluated using the CCK8 assay (Beyotime Biotechnology). Bone marrow mesenchymal stem cells (BMSCs) were seeded onto 24-well plates containing scaffolds (2 × 10^4 cells per well) in each group, with three replicates per group. After 24 h, when cells had adhered and stabilized, the ultrasound group was treated with ultrasound (40 kHz, 0.43 W/cm^2^, 20 min/day). On days 1, 3, and 7 of co-culture, the medium was replaced with the basal medium containing 10% CCK-8 solution (Beyotime Biotechnology), and the cells were incubated for an additional 1.5 h at 37°C. Then, the medium was transferred to a 96-well plate (100 µL per well) and the absorbance was measured at 450 nm by Microplate Reader (TECAN, INFINITE 200 PRO).

After 1 day of culture on the scaffolds, BMSCs were stained with FITC-labeled cyclic RGD peptide at room temperature for 60 min, followed by Hoechst 33,258 staining for 10 min. After thorough washing with PBS, the cell attachment and spreading morphology were observed under a laser confocal microscope (Leica SP8).

#### 2.3.3 *In Vitro* cell migration assay

A horizontal line was drawn on the back of 12-well plates with a marker. Scaffolds from each group were cut into circular discs (20 mm in diameter), sterilized, and placed into the 12-well plates. BMSCs were adjusted to a concentration of 2 × 10^5 cells per well and seeded into the plates. After culturing in a 5% CO2, 37°C incubator until the cell density reached approximately 90%, the cells were serum-starved for 6 h using a serum-free medium. A vertical scratch was made in each well using a 200 µL pipette tip. After washing with PBS, the medium was replaced with a fresh base culture medium. The width of the scratch was observed and photographed under a microscope. After incubation in the 37°C incubator for 24 h, Calcein-AM and PI staining were performed. After thorough washing, the scratch width was observed and recorded using a laser confocal microscope (Leica SP8) to assess cell migration.

#### 2.3.4 Alkaline phosphatase activity

BMSCs were seeded into 24-well plates preloaded with biomimetic scaffolds for each group (2 × 10^4^ cells per well), with three replicates per group. After culturing in a 37°C incubator for 24 h, the medium was replaced with Osteogenic Induction Medium (OIM), and the ultrasound group was treated with ultrasound (40 kHz, 0.43 W/cm^2^, 20 min/day). On days 7 and 14 of co-culture, alkaline phosphatase (ALP) activity in each group was evaluated using an ALP assay kit (Beyotime Biotechnology).

#### 2.3.5 Quantitative polymerase chain reaction (qPCR)

BMSCs were seeded into 24-well plates preloaded with biomimetic scaffolds for each group (2 × 10^4^ cells per well), with three replicates per group. After culturing in a 37°C incubator for 24 h, the medium was replaced with osteogenic/tendinogenic differentiation medium, and the ultrasound group was treated with ultrasound (40 kHz, 0.43 W/cm^2^, 20 min/day). At days 7 and 14 of co-culture, total RNA was extracted from each group. After determining the RNA concentration and purity using a nanodrop spectrophotometer, reverse transcription was performed. The expression levels of osteogenic marker genes OPN, OCN, and RUNX2 (Supplementary Table S1) and tendinogenic marker genes SCX and TNMD (Supplementary Table S2) were analyzed by qPCR. The relative expression levels of target genes were normalized to reference gene levels using the 2^−ΔΔCT^ method and are presented as the mean ± SD.

#### 2.3.6 *In vitro* macrophage polarization assay

The RAW 264.7 macrophages were obtained from Cell-Lab SysTech (Shanghai, China). RAW 264.7 cells were cultured in Dulbecco’s modified Eagle medium (DMEM) supplemented with 1% penicillin-streptomycin solution and 10% FBS at 37°C in an atmosphere containing 5% CO2.

RAW264.7 cells were seeded into 24-well plates pre-coated with the respective patches (2 × 10^4^ cells per well), with 3 replicate wells per group. After 24 h, when cells adhered and stabilized, the ultrasound group was treated with ultrasound (40 kHz, 0.43 W/cm^2^, 20 min/day). After 3 days of co-culture, qPCR analysis was performed to measure the expression of M1 macrophage markers iNOS and TNF-α, and M2 macrophage marker Arg-1. Additionally, an enzyme-linked immunosorbent assay (ELISA) was used to quantify the secretion of the inflammatory cytokines IL-1β and IL-10.

### 2.4 Animal experiment

#### 2.4.1 Animal model building and patch implantation

We adhered to the Animal Research: Reporting *In Vivo* Experiments (ARRIVE) 2.0 guidelines. This study was approved by the Animal Management and Use Committee of Beijing Jishuitan Hospital (Ethical Approval No: Jilun Dong Shen Zi No. 2023-07-01). A total of 36 adult male New Zealand white rabbits were randomly divided into three groups: the control group (simple suturing), the piezoelectric patch group, and the piezoelectric patch + ultrasound group. After anesthesia, the deltoid muscle was separated, the supraspinatus tendon insertion was severed, and residual tissue was removed to expose the bone marrow, thus creating a rabbit rotator cuff injury model. Ultrasound stimulation (1 MHz, 0.86 W/cm^2^, 10 min/day) was applied starting 1 day after surgery for the ultrasound group.

#### 2.4.2 MicroCT evaluation and histological staining

Specimens from the supraspinatus tendon-humeral head complex were harvested at 6 and 12 weeks post-surgery for analysis. Macroscopic observation of supraspinatus tendon healing at the humeral greater tuberosity insertion site, as well as any surrounding inflammation or adipose accumulation, was conducted. The newly formed bone at the insertion site was assessed using a Quantum FX high-resolution small animal Micro-CT scanner. Tissue samples were fixed, decalcified, embedded, sectioned, and stained with H&E, Oil Red O-Safranin O, Masson’s trichrome, and Sirius Red. Biomechanical testing of the supraspinatus tendon-humerus complex was also performed.

### 2.5 Data analysis

Data were analyzed using SPSS Statistics 27.0 and are presented as mean ± standard deviation. One-way ANOVA with Tukey’s *post hoc* test was used for statistical analysis, with a significance level set at P < 0.05. Values of *P < 0.05, **P < 0.01, or ***P < 0.001 were considered statistically significant.

## 3 Result and discussion

### 3.1 Morphology and characterization

**FIGURE 1:**
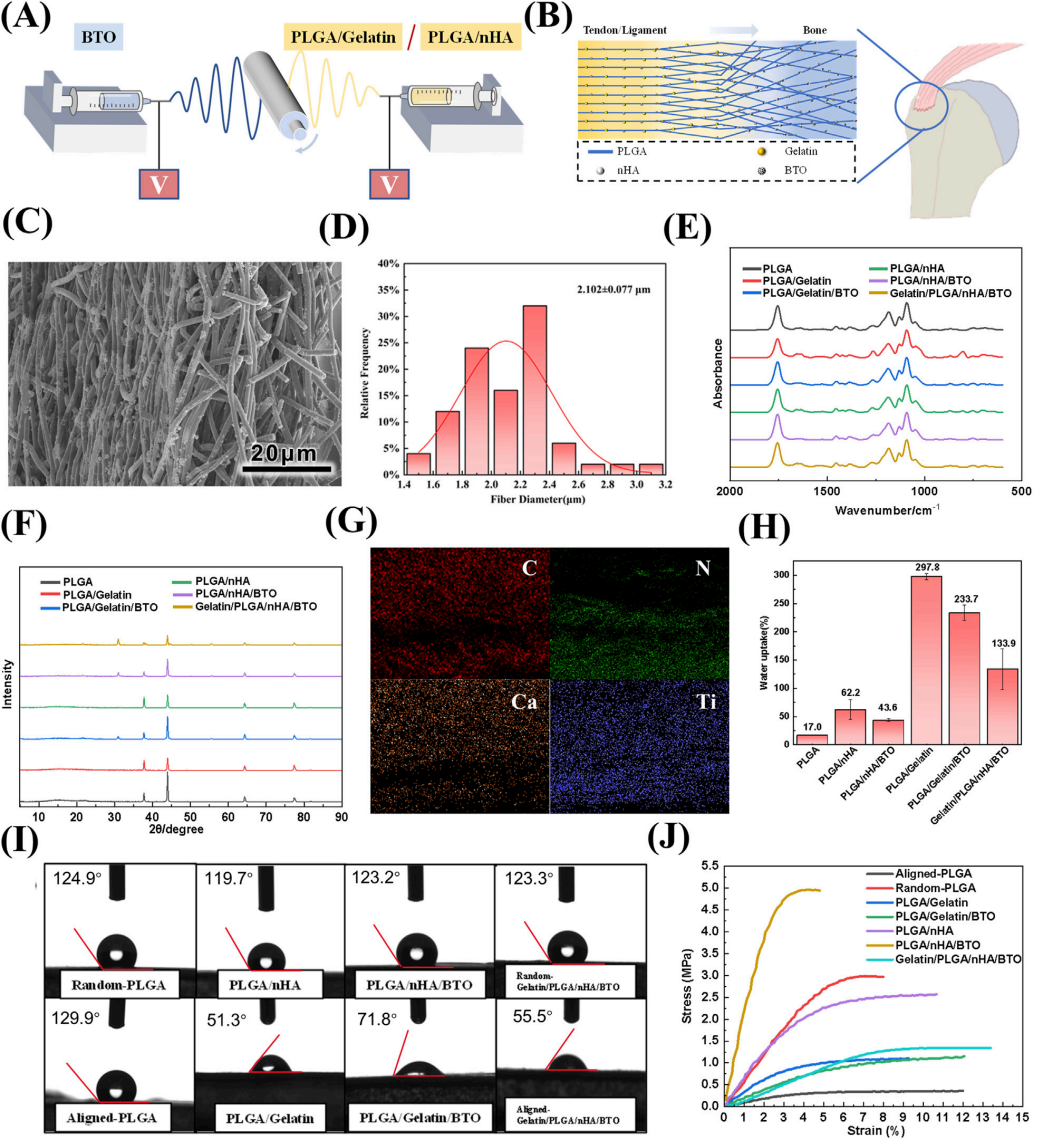
Preparation and Characterization of Biomimetic Gelatin/PLGA/nHA/BTO Piezoelectric Patches. **(A)** Dual-spinneret electrospinning apparatus for patch preparation; **(B)** Biomimetic patch simulated structure; **(C)** SEM image; **(D)** Fiber diameter of the patch; **(E)** Infrared absorption spectrum; **(F)** XRD pattern; **(G)** Elemental distribution map; **(H)** Swelling ratio; **(I)** Contact angle; **(J)** Tensile strength.

Bionic piezoelectric patches of Gelatin/PLGA/nHA/BTO were fabricated using a dual-nozzle electrospinning system, as shown in Figure 1A. The simulated morphology of the patches is shown in Figure 1B. Scanning electron microscopy (SEM) images revealed that nHA and BTO appeared as spherical particles attached to the smooth, uniform surface of the Gelatin/PLGA fibers. The cross-sectional microstructure exhibited a transition from oriented to random arrangement, achieving the desired bionic structural effect (Figure 1C; Supplementary Figure S1A). Fiber diameter analysis indicated that the final diameter of the patches was approximately 2.102 μm (Figure 1D; Supplementary Figure S1B).

Fourier-transform infrared spectroscopy (FTIR) revealed that the introduction of gelatin and hydroxyapatite did not significantly affect the positions of the diffraction peaks corresponding to the functional groups of PLGA, likely due to their low content and encapsulation within the fibers (Figure 1E; Supplementary Figure S2A–C). The X-ray diffraction (XRD) results demonstrated the presence of the primary diffraction peaks of the tetragonal crystal system of barium titanate (BTO), accompanied by the observation of a double peak phenomenon at 2θ = 45°, thereby substantiating its advantageous piezoelectric properties (see Figure 1F; Supplementary Figure S2D). Elemental distribution maps for carbon (C), nitrogen (N), calcium (Ca), and titanium (Ti) indicated that the components of the patch were tightly integrated, and a gradient distribution of nitrogen (N) and calcium (Ca) elements was observed, resembling the mineral content and collagen fibre tissue changes from tendon to bone in the rotator cuff structure (see Figure 1G; Supplementary Figure S2E).

Natural macromolecular gelatin, known for its excellent hydrophilicity, played a dominant role in the swelling behavior of the patches. The incorporation of nHA and BTO affected the surface roughness of the fibers, resulting in a lower swelling rate compared to the unmodified patch, with a final swelling rate of 133.9% (Figure 1H). The variations in contact angle after the addition of each component exhibited a consistent trend with the swelling ratio (Figure 1I). The random and aligned layers of the patch demonstrated contact angles of 123.2° ± 0.7° and 55.5° ± 4.4°, respectively, indicating that the aligned layer possesses significantly higher hydrophilicity. This phenomenon arises from the smoother surface of the aligned fiber membrane (with lower anisotropic roughness), which reduces air entrapment beneath the droplet (i.e., diminishing the Cassie-Baxter superhydrophobic state) and facilitates liquid infiltration into the surface (transitioning to the Wenzel wetting state) (Liao et al., 2024; Zhai et al., 2024). Due to the reinforcing effect of nanoparticles within the polymer matrix, the incorporation of gelatin, hydroxyapatite, and barium titanate improved the ultimate strength of the original PLGA. The fracture strength of the bionic Gelatin/PLGA/nHA/BTO piezoelectric patch was 1.34 ± 0.08 MPa, demonstrating favorable biomechanical properties (Figure 1J).

**FIGURE 2:**
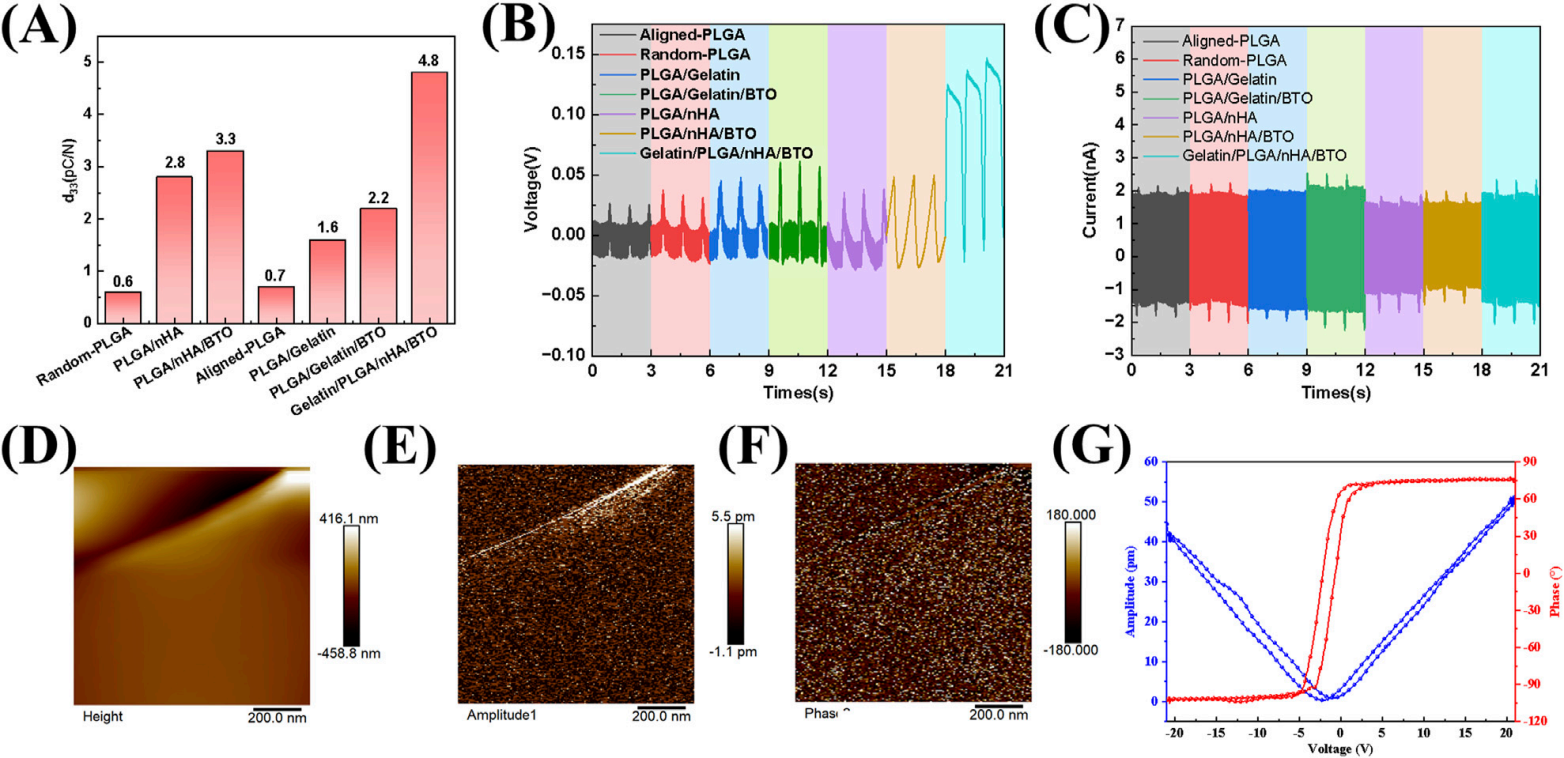
Characterization of the piezoelectric properties of the biomimetic Gelatin/PLGA/nHA/BTO piezoelectric patch. **(A)** Piezoelectric coefficient; **(B)** Open-circuit voltage; **(C)** Short-circuit current; **(D)** Surface morphology of the piezoelectric force microscopy; **(E)** Piezoelectric amplitude map; **(F)** Piezoelectric phase map; **(G)** Voltage-amplitude curve and voltage-phase curve.

The piezoelectric coefficient d33 of the biomimetic piezoelectric patch Gelatin/PLGA/nHA/BTO was measured to be 4.8 pC/N, which results from the combined effects of gelatin, hydroxyapatite (nHA), and barium titanate (BTO). Gelatin, a product of collagen hydrolysis, has lost part of its three-dimensional helical structure, but its piezoelectricity remains higher than that of PLGA (Fan et al., 2024). Nano-hydroxyapatite, as the main mineral phase of bone, inherently possesses some piezoelectric properties (Ahn and Grodzinsky, 2009; Heng et al., 2023). BTO, with its tetragonal crystal structure, exhibits high asymmetry and can undergo spontaneous polarization (Suzana et al., 2023; Li S. et al., 2025). Under mechanical stress, it is more prone to generating electrical signals, thus demonstrating excellent piezoelectric properties and significantly enhancing the piezoelectricity of electrospun rotator cuff patches. Moreover, the interaction between BTO and hydroxyapatite can generate a higher piezoelectric potential, improving the piezoelectric characteristics of the patch (Figure 2A). Open-circuit voltage and short-circuit current of the patch were measured using an electrometer. A 1 Hz, 20 N stable and uniform impact force was applied to the rotator cuff patch by a modal shaker, with a force area of 4 cm^2^. The observed results were consistent with the piezoelectric coefficient d33 data (Figures 2B,C). The patch’s microscopic piezoelectric effect was characterized using a piezoresponse force microscope (PFM), where a “butterfly curve” was observed. The curve was symmetric at about 0 V, with minimal offset, and at a voltage of approximately ±5 V, a 180° phase reversal occurred, indicating that the biomimetic piezoelectric patch Gelatin/PLGA/nHA/BTO exhibited good piezoelectric and ferroelectric properties, meeting the performance requirements for repair (Figures 2D–G).

### 3.2 *In vitro* cell viability and migration

**FIGURE 3:**
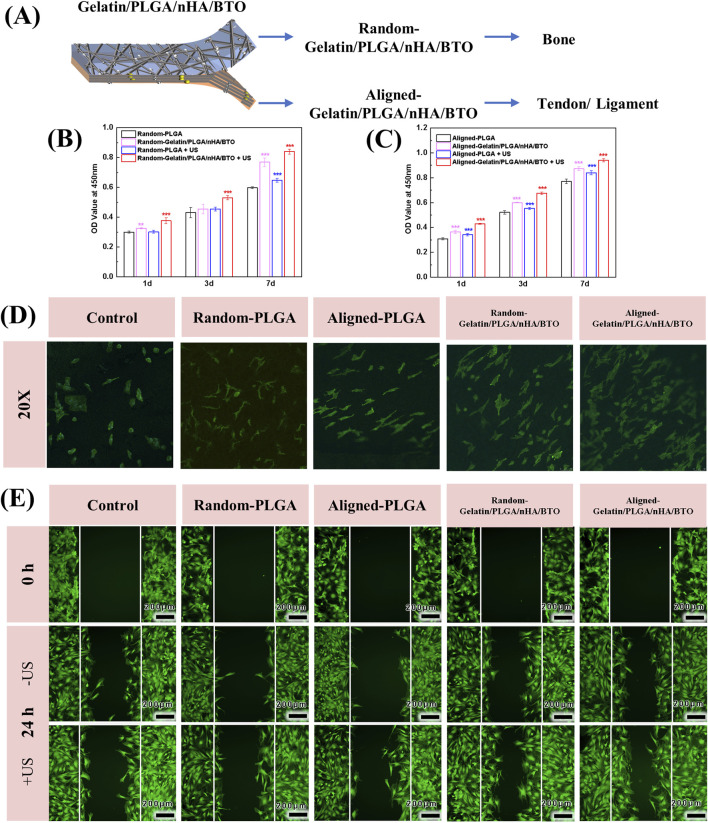
*In vitro* cell viability and migration. **(A)** Cell culture groups; **(B)** Random side cell proliferation; **(C)** Aligned side cell proliferation; **(D)** CLSM images of cell proliferation; **(E)** CLSM images of cell migration. All data represent mean ± SD (n ≥ 3). *P < 0.05 vs. time-matched PLGA, **P < 0.01 vs. time-matched PLGA, ***P < 0.001 vs. time-matched PLGA.

To evaluate the *in vitro* biocompatibility of the biomimetic piezoelectric rotator cuff patch Gelatin/PLGA/nHA/BTO, BMSCs were seeded on both sides of the patch (Figure 3A). The effect of the patch on BMSC proliferation was assessed using the CCK-8 assay (Figures 3B,C). As the co-culture time of the cell-patch complex increased, the absorbance values of both the random and aligned sides of the biomimetic piezoelectric patch showed a significant increase compared to the control group. Additionally, ultrasonic activation stimulated cell growth and promoted cell proliferation. CLSM images showed that after co-culturing rat BMSCs with the random and aligned surfaces of the biomimetic piezoelectric patch, the cells exhibited good morphology and various degrees of elongation, indicating that the Gelatin/PLGA/nHA/BTO biomimetic piezoelectric patch had no apparent toxicity and did not affect the normal morphology of the cells, demonstrating good biocompatibility (Figure 3D).

*In vitro*, cell migration experiments showed that the cell migration rate in the biomimetic piezoelectric patch group was enhanced, with a wound healing rate of approximately 50%. In the group exposed to ultrasonic irradiation, most of the migrated BMSCs covered the wound area, with the wound area change reaching 62%, whereas other groups still showed a large wound width. This indicates that the Gelatin/PLGA/nHA/BTO biomimetic piezoelectric patch can accelerate cell migration, and the effect is more pronounced after ultrasound activation (Figure 3E).

### 3.3 *In Vitro* cellular differentiation

**FIGURE 4:**
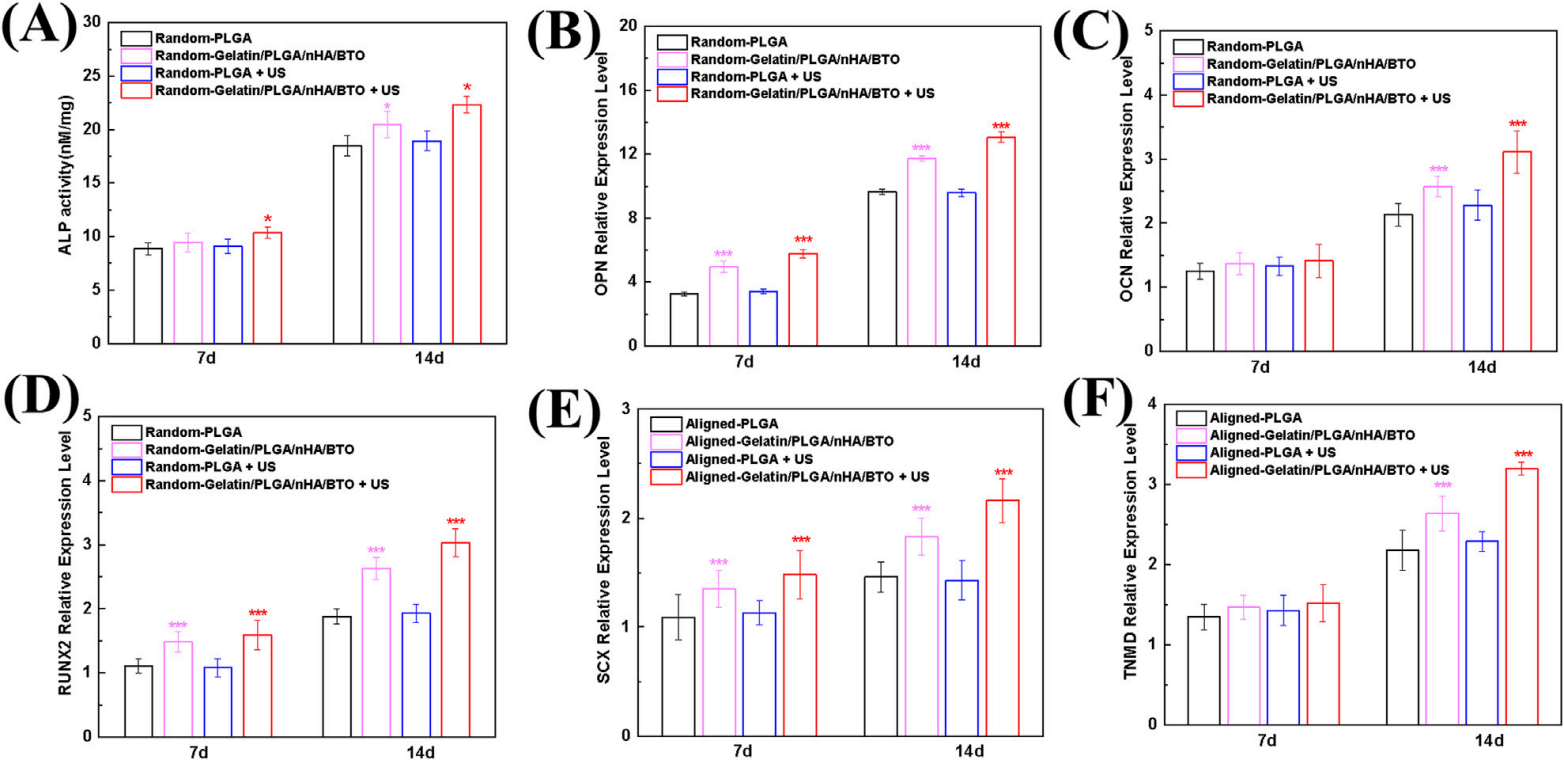
*In vitro* Cellular Differentiation **(A)** ALP activity during osteogenic differentiation; **(B)** OPN gene expression during osteogenic differentiation; **(C)** OCN gene expression during osteogenic differentiation; **(D)** RUNX2 gene expression during osteogenic differentiation; **(E)** SCX gene expression during tenogenic differentiation; **(F)** TNMD gene expression during tenogenic differentiation. All data represent mean ± SD (n ≥ 3). *P < 0.05 vs. time-matched PLGA, **P < 0.01 vs. time-matched PLGA, ***P < 0.001 vs. time-matched PLGA.

The osteogenic differentiation of the scaffold under ultrasonic stimulation was evaluated by measuring ALP activity (Figure 4A). The results show that the scaffold group under ultrasound stimulation exhibited the highest ALP activity, indicating that ultrasound activation of the biomimetic piezoelectric scaffold effectively promotes osteogenic differentiation. Osteopontin (OPN), a secreted phosphoprotein synthesized by osteoblasts, belongs to the Small Integrin-Binding Ligand N-linked Glycoprotein (SIBLING) family and serves as a critical regulator of bone matrix mineralization, osteoblast-osteoclast cross-talk, and calcium homeostasis during skeletal maturation (Li X. et al., 2025). Runt-related transcription factor 2 (RUNX2), a master transcriptional activator, governs the osteogenic commitment of bone marrow-derived mesenchymal stem cells (BMSCs) through direct binding to osteoblast-specific cis-regulatory elements (e.g., OCN, BSP promoters) (Gargalionis et al., 2024). Further analysis of the expression of osteogenic differentiation-related genes, including OPN, OCN, and RUNX2, validated the scaffold’s osteogenic promotion effect (Figures 4B–D). The biomimetic piezoelectric scaffold likely initiates early gene expression of OPN and RUNX2, with significant expression observed as early as day 7 of culture, and the effect was enhanced upon ultrasound activation. The normal expression time of the OCN gene corresponds to the mid-to-late stage of osteogenic differentiation, and on day 14 of culture, the relative expression level of OCN significantly increased, with the ultrasound-activated biomimetic piezoelectric scaffold group showing the highest expression. This suggests that the non-oriented scaffold promotes bone formation and has enhanced osteogenic potential when activated by ultrasound. Emerging studies have demonstrated that piezoelectric scaffolds can promote osteogenic differentiation of osteoblasts through the generation of low-intensity electrical cues. Furthermore, electrical stimulation (ES) activates the Ca^2+^/CaMKII/CREB signaling axis, triggering calcitonin gene-related peptide (CGRP) biosynthesis and pulsatile release. This neuropeptide-mediated mechanism enhances bone defect repair by coupling angiogenesis and osteoclast-osteoblast coupling (Li X. et al., 2025; Wu et al., 2023; Mi et al., 2022).

Tendon lineage markers Scleraxis (Scx), a basic helix-loop-helix transcription factor, and Tenomodulin (TNMD), a type II transmembrane glycoprotein, orchestrate tenogenic differentiation by coordinating extracellular matrix (ECM) remodeling (via MMP2/9 modulation) and mechanoresponsive signaling pathways (YAP/TAZ activation) (Yamamoto et al., 2022). The tendon differentiation-promoting effect of the oriented scaffold under ultrasound stimulation was verified by analyzing SCX and TNMD, tendon differentiation-related genes (Figures 4E,F). The results show that the biomimetic piezoelectric scaffold promoted SCX gene expression as early as day 7 of culture, with the promotion effect significantly enhanced after ultrasound activation. The relative expression level of TNMD exhibited a similar trend on day 14. These findings indicate that the oriented scaffold promotes tendon formation, and its tendonogenic potential is enhanced upon ultrasound activation, consistent with the results for osteogenic differentiation. Existing studies have proved that calcium activity and endogenous electric fields related to tissue damage can reactivate the signaling pathways involved in embryonic development, thereby promoting tissue regeneration. Moreover, tendon cells can sense environmental mechanical and electrical signals and convert them into the activation of downstream signaling pathways, thereby regulating their functions and participating in the repair process at the tissue level (Fernandez-Yague et al., 2021).

### 3.4 *In vitro* inflammatory regulation

**FIGURE 5:**
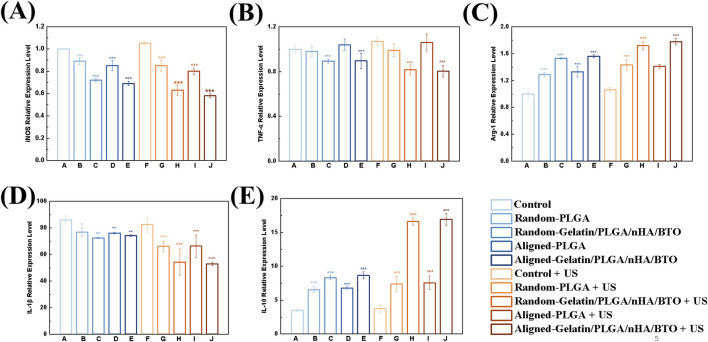
*In vitro* inflammation regulation. **(A)** iNOS expression level; **(B)** TNF-α expression level; **(C)** Arg-1 expression level; **(D)** IL-1β expression level; **(E)** IL-10 expression level. All data represent mean ± SD (n ≥ 3). *P < 0.05 vs. control, **P < 0.01 vs. control, ***P < 0.001 vs. control.

The effects of the patch on macrophage polarization were assessed by analyzing the expression of M1 macrophage marker genes iNOS and TNF-α, as well as the M2 macrophage marker gene Arg-1 (Figures 5A–C). In the absence of ultrasound treatment, the expression of iNOS and TNF-α in macrophages co-cultured with the patch was suppressed, while the expression of Arg-1 was enhanced. After ultrasound activation, the expression of M1 marker genes was further inhibited, while the expression of M2 marker genes was further increased. Further analysis of the secretion of the inflammatory cytokines IL-1β and IL-10 revealed the following (Figures 5D,E). Without ultrasound treatment, the secretion of the pro-inflammatory cytokine IL-1β was reduced, while the secretion of the anti-inflammatory cytokine IL-10 was increased in the macrophages co-cultured with the patch. Following ultrasound activation, the secretion of pro-inflammatory IL-1β was significantly reduced, while the secretion of anti-inflammatory IL-10 was significantly increased, consistent with the gene expression results. Local inflammation and the immune microenvironment play a significant role in bone homeostasis and healing. Macrophages are pivotal in immune regulation due to their high degree of plasticity, which typically leads to their classification into pro-inflammatory (M1) and anti-inflammatory (M2) subtypes based on their functions (Wu et al., 2023). Macrophages are electrophysiologically active cells capable of responding to electric fields, which promote electrical conduction and aid in the repair of tissues such as myocardium, skin, and bone. Macrophage-mediated immune regulation can result in sustained responses to implants by secreting a range of anti-inflammatory cytokines and other mediators, while inhibiting the secretion of pro-inflammatory cytokines, thereby modulating macrophage polarization (downregulating the pro-inflammatory M1 phenotype and/or upregulating the anti-inflammatory M2 phenotype) (Sun et al., 2023). It suggests that local electrical stimulation from the bionic piezoelectric patch (Gelatin/PLGA/nHA/BTO) activated by ultrasound can significantly promote M2 macrophage polarization, which is more favorable for cellular repair and regeneration. Studies have shown that ultrasound-induced piezoelectric stimulation promotes Ca 2+ influx by activating transient receptor potential vanalic acid channel 1 (TRPV1), thereby activating the cAMP signaling pathway, and ultimately achieving anti-inflammatory and tissue repair promotion effects (Huang et al., 2025; Shi et al., 2024).

### 3.5 *In-Vivo* rotator cuff repair

A rabbit supraspinatus tendon injury model was established (Supplementary Figure S3A). The experimental rabbits in all three groups remained in good condition, with no signs of functional impairment, wound infection, loss of appetite, or weight loss. The wounds exhibited a robust recovery process. Upon examination of the right shoulder surgical region and surrounding tissues, it was observed that the rotator cuff structures in all three groups were intact, with the repaired supraspinatus tendon closely connected to the greater tuberosity of the humerus, with no displacement or rupture, and no signs of inflammatory responses in the surrounding tissue (Supplementary Figure S3B).

**FIGURE 6:**
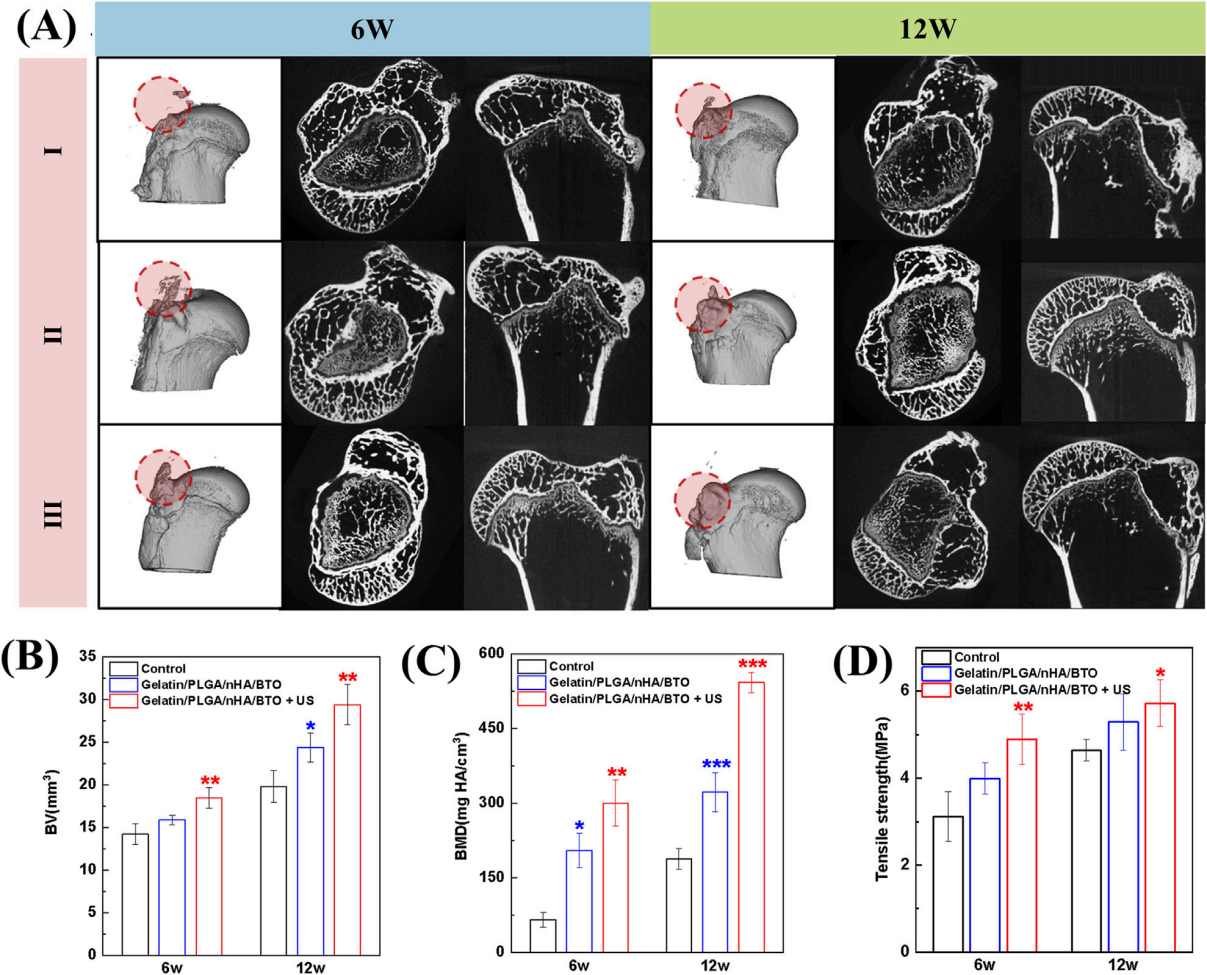
Tendon-bone interface Micro-CT images, quantitative analysis, and biomechanical analysis. **(A)** Micro-CT images (I: Control group; II: Gelatin/PLGA/nHA/BTO group; III: Gelatin/PLGA/nHA/BTO + US group); **(B)** Bone volume; **(C)** Bone density; **(D)** Biomechanical analysis. All data represent mean ± SD (n ≥ 3). *P < 0.05 vs. control, **P < 0.01 vs. control, ***P < 0.001 vs. control.

Three-dimensional reconstructions of the groups are shown in Figure 6A. At 6 and 12 weeks, both the Gelatin/PLGA/nHA/BTO and Gelatin/PLGA/nHA/BTO + US groups exhibited more newly formed bone tissue at the tendon-bone interface, with the bone being relatively dense after patch implantation. At 12 weeks post-surgery, a substantial amount of new bone formation was observed at the injury site in all groups, with the group receiving the piezoelectric patch showing more complete and dense new bone. Furthermore, the new bone volume (BV) and bone mineral density (BMD) at the tendon-bone interface were quantitatively analyzed (Figures 6B,C), showing consistent trends across the groups. The implantation of the bioinspired piezoelectric patch Gelatin/PLGA/nHA/BTO significantly increased the bone volume and density of the newly formed bone at the injury site, with further enhancement following ultrasound activation of the piezoelectric patch. The quantitative analysis results were consistent with the CT three-dimensional reconstruction images.

The native tendon-bone interface enables gradual stress dissipation from compliant tendon to rigid bone through its fibrocartilaginous enthesis, effectively mitigating interfacial stress concentration via its graded composition. However, post-traumatic scar healing in rotator cuff injuries results in mechanically incompetent fibrovascular tissue formation, creating abrupt stiffness transitions that localize tensile stresses at the repair site. This biomechanical mismatch predisposes to recurrent tearing. Consequently, reconstructing the hierarchical architecture of the tendon-bone junction–particularly restoring its tri-layered transitional structure (tendon → fibrocartilage → calcified cartilage) is recognized as a critical objective in next-generation biomaterial design (Li X. et al., 2025). The tensile strength of the supraspinatus tendon-humerus complex after rotator cuff repair was assessed (Figure 6D). From 6 to 12 weeks post-surgery, the tensile strength increased in all groups, indicating that the tendon-bone interface healing was not yet complete at 6 weeks post-surgery. The Gelatin/PLGA/nHA/BTO + US group demonstrated the highest tensile strength, confirming that the piezoelectric patch, when activated by ultrasound, exhibited superior *in vivo* repair performance. This further validates that the piezoelectric patch Gelatin/PLGA/nHA/BTO can induce new bone formation at the tendon-bone interface, promote integration between the new bone and tendon, and significantly enhance the bone regeneration capacity at the tendon-bone interface after ultrasound activation.

**FIGURE 7:**
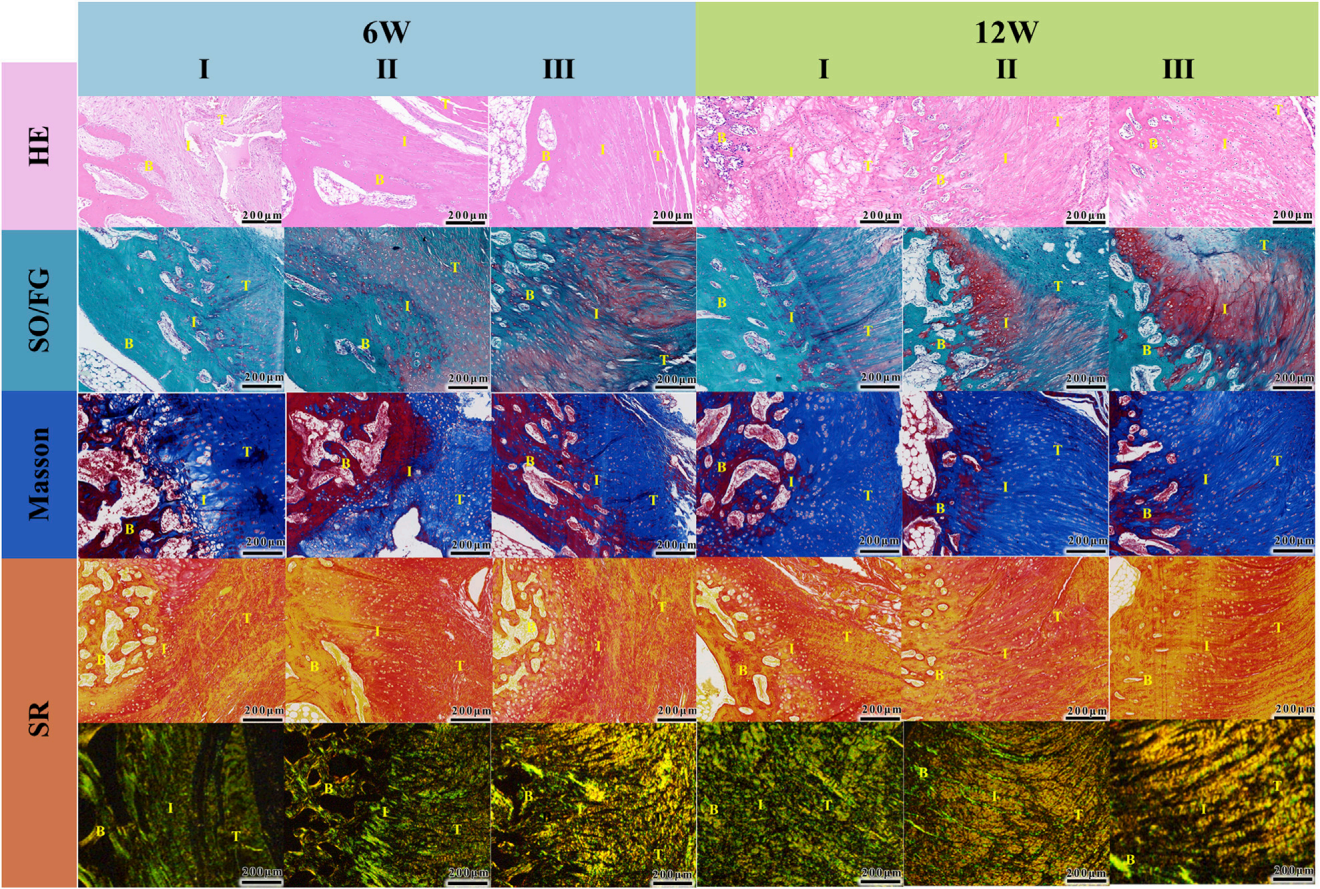
Histological analysis (I: Control; II: Gelatin/PLGA/nHA/BTO; III: Gelatin/PLGA/nHA/BTO + US). From top to bottom: HE staining, Orange G-fast green staining, Masson staining, Sirius Red staining (observed under optical microscope and polarized light). T-Tendon; B-Bone; I,: Tendon-Bone Interface. All data represent mean ± SD (n ≥ 3).

The supraspinatus tendon-humerus complex specimens were subjected to histological analysis (see Figure 7). Hematoxylin and eosin (HE) staining revealed that at 6 weeks, the graft had begun to be absorbed, and the tissue at the repair interface started to align in an organized manner. The number of fibroblasts decreased, and collagen fibers grew in an orderly fashion. By the 12th week, no significant residual piezoelectric graft material was observed. Modified Safranin O-fast green (SO/FG) staining differentiated cartilage and bone tissues, with cartilage stained red, which was used to assess the regeneration of fibrocartilage at the tendon-bone interface of the rotator cuff. The Gelatin/PLGA/nHA/BTO + US group exhibited the largest area of fibrocartilage metachromasia, and at 12 weeks, both tendon-bone interfaces in the grafted groups were connected by new mineralized tissue. Masson staining was used to observe collagen fiber formation at the rotator cuff tendon-bone interface. At 6 weeks post-surgery, collagen fibers were generated at the tendon-bone interface in all groups, but the arrangement was disordered. By the 12th week, the number of collagen fibers had increased, with Gelatin/PLGA/nHA/BTO and Gelatin/PLGA/nHA/BTO + US groups showing more regular collagen fiber alignment, especially in the Gelatin/PLGA/nHA/BTO + US group, where the fibers were more densely packed. Sirius Red staining was used to differentiate collagen types; under bright-field microscopy, collagen fibers appeared red, while other tissue components were stained yellow. Under polarized light, different collagen fibers exhibited varying optical properties, and type I collagen fibers appeared yellow or red. Under bright-field microscopy, at 6 weeks, collagen fiber generation was observed at the tendon-bone interface in all groups, with the fibers arranged in a disordered manner. The two grafted groups had significantly more collagen fibers than the control group. By the 12th week, the collagen fiber count increased in all groups, with the Gelatin/PLGA/nHA/BTO + US group showing the most densely and orderly arranged collagen fibers. Under polarized light, at both 6 and 12 weeks, the new collagen fiber bundles at the tendon-bone interface in the Gelatin/PLGA/nHA/BTO + US group were most orderly arranged, forming a continuous and parallel fiber structure along the interface, while the control group showed continuous, irregular fibrous tissue at the interface. These findings suggest that both the Gelatin/PLGA/nHA/BTO and Gelatin/PLGA/nHA/BTO + US groups exhibited greater type I collagen production, with the piezoelectric graft combined with ultrasound showing the most superior results. This demonstrates that the bionic piezoelectric graft Gelatin/PLGA/nHA/BTO, under ultrasound stimulation, can promote bone and cartilage regeneration at the rotator cuff tendon-bone interface, enhance collagen fiber maturation and alignment, and particularly promote the generation of type I collagen with high biomechanical strength, effectively facilitating rotator cuff tendon-bone healing *in vivo*.

## 4 Conclusion

This study developed a bionic piezoelectric patch Gelatin/PLGA/nHA/BTO with a compositional gradient structure, incorporating both aligned and non-aligned fiber configurations, using a dual-nozzle electrospinning technique. The patch has been developed for the purpose of repairing rotator cuff injuries in the presence of ultrasound stimulation. In comparison to conventional rotator cuff patches, the following properties are exhibited: i) a biomimetic structure that simulates the rotator cuff tendon-bone interface, thereby promoting layered repair of different tissues; ii) excellent piezoelectric properties, which provide a suitable microenvironment for biological repair under ultrasound stimulation; and iii) enhanced macrophage polarization, anti-inflammatory factor generation, and modulation of the immune microenvironment, thus effectively promoting tissue regeneration under ultrasound. This research provides important insights for the design and future development of intelligent functionalized biomaterials and their application in rotator cuff interface tissue engineering. However, the specific mechanisms of smart electrostimulation biomaterials require further detailed investigation. Moreover, in practical biomedical applications, more research is needed to precisely and appropriately control smart electrostimulation in rotator cuff repair and regeneration. This remains a clear long-term research focus with significant implications for the clinical application of novel intelligent biomaterials that promote rotator cuff tendon-bone interface regeneration.

## Data Availability

The original contributions presented in the study are included in the article/Supplementary Material, further inquiries can be directed to the corresponding author.
