# Supplementary material for: Bioinspired piezoelectric patch design for sonodynamic therapy: a preclinical mechanistic evaluation of rotator cuff repair and functional regeneration
Source: Front Bioeng Biotechnol. 2025 May 21;13:1565347. doi: 10.3389/fbioe.2025.1565347 (PMC12133869; doi:10.3389/fbioe.2025.1565347)
Supplement: Supplementary file 1 [file DataSheet1.docx]

Supplementary Material
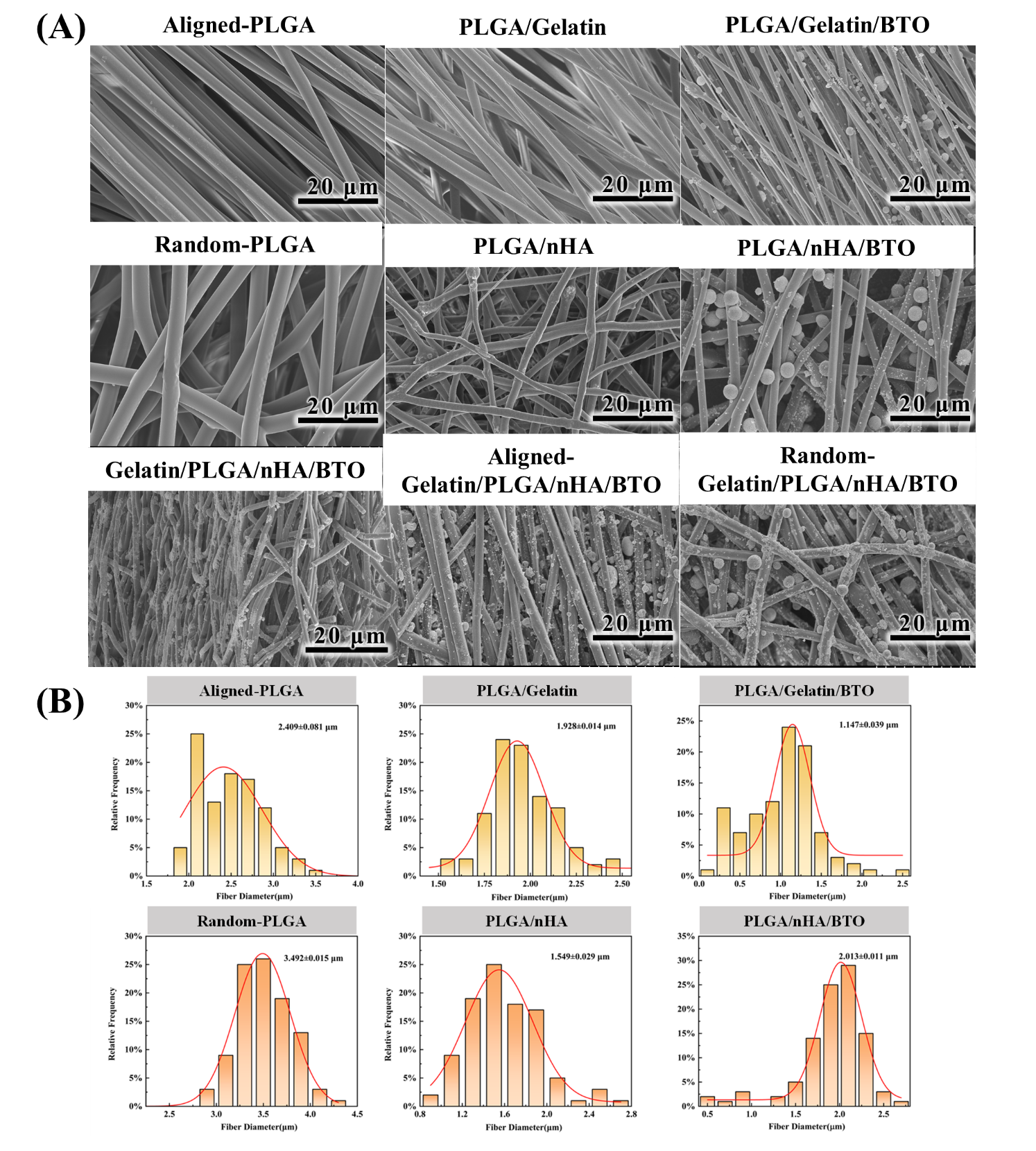


Figure S1：Characterization of Biomimetic Gelatin/PLGA/nHA/BTO Piezoelectric Patches. (a) SEM image; (b)Fiber diameter of the patch


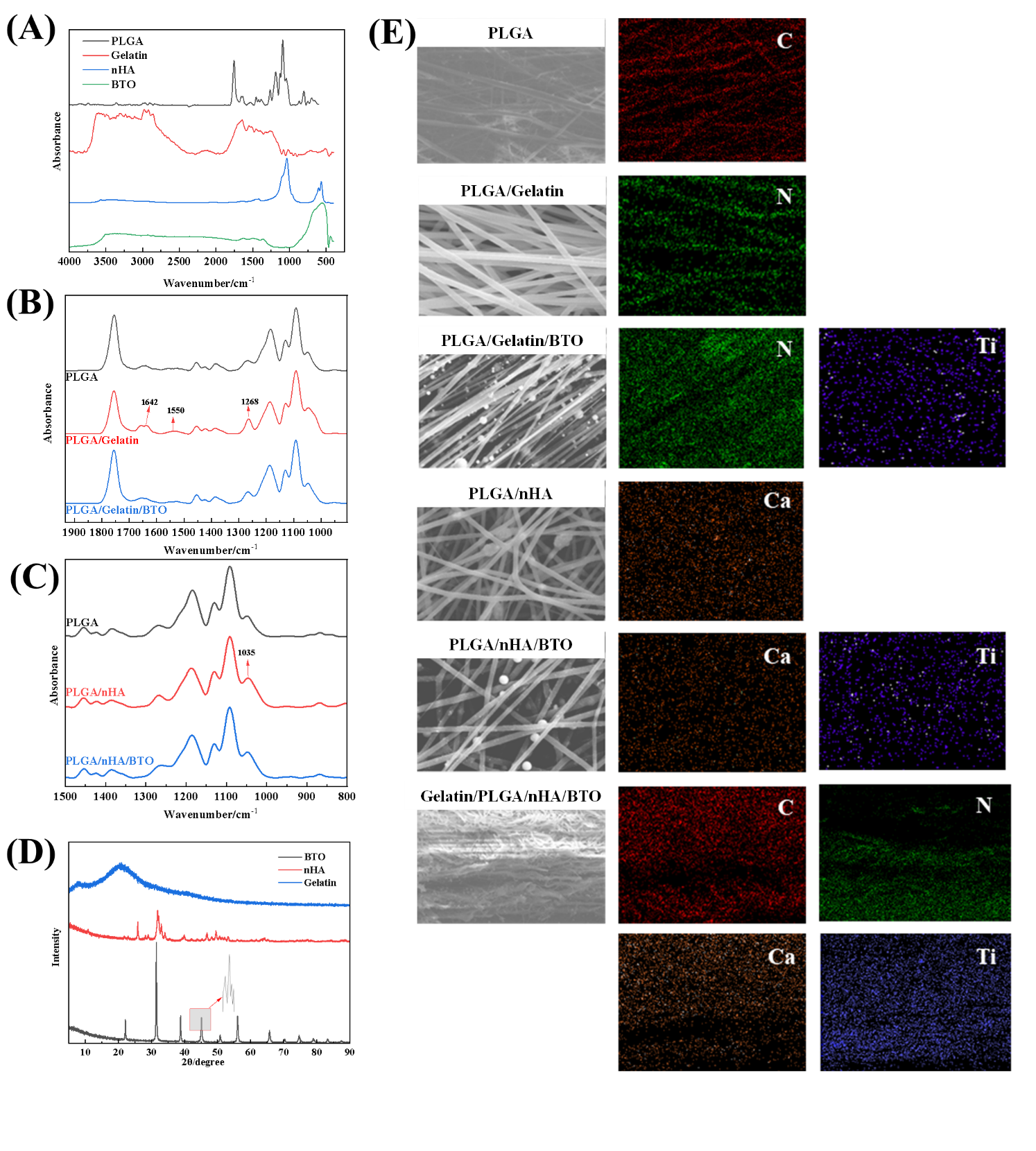


Figure S2：Characterization of Biomimetic Gelatin/PLGA/nHA/BTO Piezoelectric Patches. (a-c) Infrared absorption spectrum; (d)XRD pattern; (e)Elemental distribution map


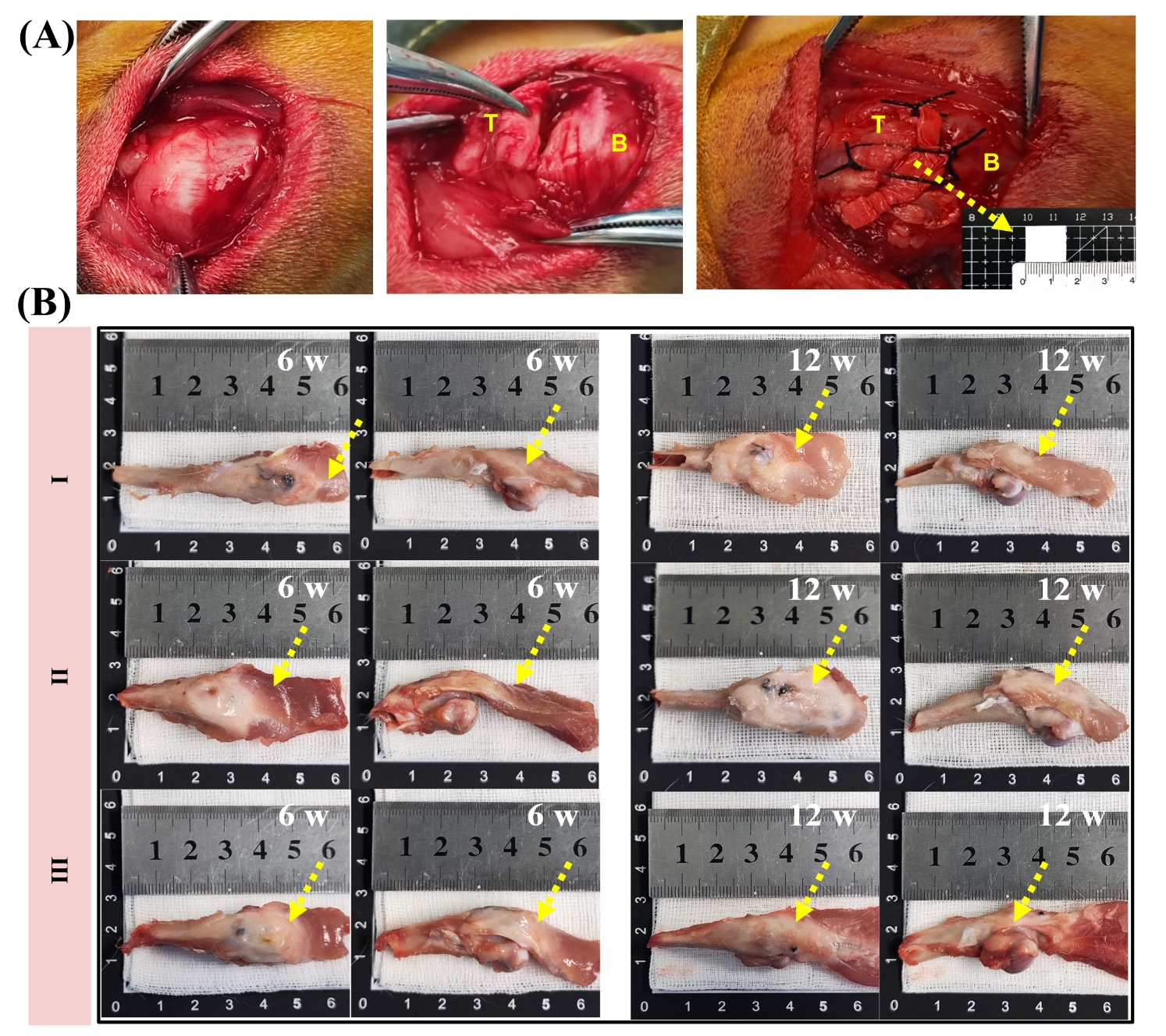


Figure S3：Macroscopic view of in vivo rotator cuff repair outcomes. (a) Surgical site and patch implantation in animal experiments; (c) Macroscopic view of the rotator cuff tendon-bone complex (I: Control group; II: Gelatin/PLGA/nHA/BTO group; III: Gelatin/PLGA/nHA/BTO+US group).

Table S1: qPCR primer sequences targeting osteogenesis-related markers

| Primer Name | Primer Sequence |
| --- | --- |
| OPN-F | TTGGCTTTGCAGTCTCCTGCGG |
| OPN-R | AGGCAAGGCCGAACAGGCAAA |
| OCN-F | CAGTAAGGTGGTGAATAGACT |
| OCN-R | GGTGCCATAGATGCGCTTG |
| RUNX2-F | TCTTCCCAAAGCCAGAGCG |
| RUNX2-R | TGCCATTCGAGGTGGTCG |
| β-actin-F | GGCTGTATTCCCCTCCATCG |
| β-actin-R | CCAGTTGGTAACAATGCCATGT |

Table S2: qPCR primer sequences targeting tendon-specific markers

| Primer Name | Primer Sequence |
| --- | --- |
| SCX-F | AACACGGCCTTCACTGCGCTG |
| SCX-R | CAGTAGCACGTTGCCCAGGTG |
| TNMD-F | TGGTGAAGACCTTCACTTTCC |
| TNMD-R | TTAAACCCTCCCCAGCATGC |
| β-actin-F | GGCTGTATTCCCCTCCATCG |
| β-actin-R | CCAGTTGGTAACAATGCCATGT |

Table S3: qPCR primer sequences targeting macrophage polarization markers

| Primer Name | Primer Sequence |
| --- | --- |
| iNOS-F | CAGCCACCTTGGTGAAGGGA |
| iNOS-R | CAACGTTCTCCGTTCTCTTGC |
| TNF-α-F | CCCTCACACTCAGATCATCTTCT |
| TNF-α-R | GCTACGACGTGGGCTACAG |
| Arg-1-F | TGATCAACTATGTCACTATGCCT |
| Arg-1-R | CGTTGAGTTCCGAAGCAAGC |
| β-actin-F | GGCTGTATTCCCCTCCATCG |
| β-actin-R | CCAGTTGGTAACAATGCCATGT |
